# Image-Guided Adaptive Brachytherapy (IGABT) for Primary Vaginal Cancer: Results of the International Multicenter RetroEMBRAVE Cohort Study

**DOI:** 10.3390/cancers13061459

**Published:** 2021-03-23

**Authors:** Henrike Westerveld, Maximilian P. Schmid, Remi A. Nout, Cyrus Chargari, Bradley R. Pieters, Carien L. Creutzberg, Alina Sturdza, Jacob C. Lindegaard, Zdenko van Kesteren, Renaud Mazeron, Nicole Nesvacil, Lars U. Fokdal

**Affiliations:** 1 Department of Radiation Oncology, Amsterdam University Medical Centers, location AMC, University of Amsterdam, 1105 Amsterdam, The Netherlands; b.r.pieters@amsterdamumc.nl (B.R.P.); z.vankesteren@amsterdamumc.nl (Z.v.K.); 2 Department of Radiation Oncology, Comprehensive Cancer Center, Medical University of Vienna, 1090 Vienna, Austria; maximilian.schmid@akhwien.at (M.P.S.); alina.sturdza@akhwien.at (A.S.); nicole.nesvacil@akhwien.at (N.N.); 3 Department of Radiation Oncology, Leiden University Medical Center, 2333 Leiden, The Netherlands; r.nout@erasmusmc.nl (R.A.N.); C.L.Creutzberg@lumc.nl (C.L.C.); 4 Department of Radiotherapy, Erasmus MC Cancer Institute, University Medical Center Rotterdam, 3015 Rotterdam, The Netherlands; 5 Brachytherapy Unit, Gustave Roussy Cancer Campus, Université Paris Saclay, 94805 Villesuif, France; cyrus.chargari@gustaveroussy.fr; 6 Department of Oncology, Aarhus University Hospital, 8200 Aarhus, Denmark; jacolind@rm.dk (J.C.L.); larsfokd@rm.dk (L.U.F.)

**Keywords:** brachytherapy, IGABT, image-guided brachytherapy, MRI, radiotherapy, vaginal cancer, RetroEMBRAVE

## Abstract

**Simple Summary:**

Primary vaginal cancer is a rare disease and, consequently, evidence about the outcome of treatment is scarce. The aim of our retrospective, observational multicenter study was to assess the oncological outcome of the nowadays standing treatment for vaginal cancer, namely radio(chemo)therapy, followed by image-guided adaptive brachytherapy (IGABT). Our study confirms the results of the earlier published small monocentric IGABT studies, showing a high local control with acceptable morbidity. Notably, patients with large (T3/T4) tumors especially seem to benefit from volumetric (3D) image-guided brachytherapy, as compared to two-dimensional-based radiotherapy. In addition, although interpreted with caution, as for cervical cancer, a higher dose seems to lead to better local control. These results should, however, be further investigated in a prospective trial.

**Abstract:**

Purpose: This study assessed outcomes following the nowadays standing treatment for primary vaginal cancer with radio(chemo)therapy and image-guided adaptive brachytherapy (IGABT) in a multicenter patient cohort. Methods: Patients treated with computer tomography (CT)–MRI-assisted-based IGABT were included. Retrospective data collection included patient, tumor and treatment characteristics. Late morbidity was assessed by using the CTCAE 3.0 scale. Results: Five European centers included 148 consecutive patients, with a median age of 63 years. At a median follow-up of 29 months (IQR 25–57), two- and five-year local control were 86% and 83%; disease-free survival (DFS) was 73% and 66%, and overall survival (OS) was 79% and 68%, respectively. Crude incidences of ≥ grade-three urogenital, gastro-intestinal and vaginal morbidity was 8%, 3% and 8%, respectively. Lymph node metastasis was an independent prognostic factor for disease-free survival (DFS). Univariate analysis showed improved local control in patients with T2–T4 tumors if >80 Gy EQD2_α/β10_ was delivered to the clinical target volume (CTV) at the time of brachytherapy. Conclusions: In this large retrospective multicenter study, IGABT for primary vaginal cancer resulted in a high local control with acceptable morbidity. These results compared favorably with two-dimensional (2D) radiograph-based brachytherapy and illustrate that IGABT plays an important role in the treatment of vaginal cancer.

## 1. Introduction

Primary vaginal cancer (PVC) is a rare gynecological cancer with an annual incidence of 0.8–1.0/100,000 women, corresponding to less than 3% of all gynecological malignancies [1]. The etiology resembles primary cervical cancer, with human papilloma virus (HPV) being the main etiological factor [2].

Treatment of vaginal cancer includes an organ-sparing approach with pelvic external beam radiotherapy (EBRT), followed by a brachytherapy (BT) boost to the residual tumor, to achieve a high dose with maximum sparing of the organs of risk (OAR)[3]. In the case of a locally advanced tumor (≥T2), radiotherapy is preferably combined with concomitant chemotherapy with weekly cisplatin [4,5].

During the last decades, new imaging techniques have been adopted in radiotherapy. For EBRT, these developments include the use of computer tomography (CT) and/or magnetic resonance imaging (MRI) for contouring of the target and organs at risk (OAR), for highly conformal treatment planning and image guidance during treatment. These techniques have led to a decrease in dose delivered to the organs at risk, and consequently reduction of acute and late morbidity [6,7].

For brachytherapy similar improvements have taken place with the introduction of image-guided adaptive brachytherapy (IGABT). The principle of IGABT is to apply repetitive three-dimensional (3D) imaging (preferably with MRI), at minimum before EBRT and at time of BT, and combine these images with gynecological examination and define different target volumes according to their presumed tumor density meanwhile taking the tumor regression during EBRT into account [8]. In locally advanced cervical cancer (LACC), improvements have enabled tumor specific dose escalation resulting in an improved local control, with simultaneous reduction of late morbidity [9].

Vaginal cancer is a rare disease, and therefore only retrospective and mainly monocentric studies with a limited number of patients are available [3]. In the historic series, most studies report on the results of patients treated with two-dimensional (2D) radiograph-based brachytherapy. In addition, recently a few small (patient numbers < 25) studies reported outcomes following MRI-based IGABT. Although some of the larger radiograph-based studies showed good results, especially in the patients treated for small tumors, the few 3D-based studies showed promising results with a high local control, not only in the small but also in the more advanced tumors [3].

In 2013 the Gynaecological Working Group of the Groupe Européen de Curiethérapie and the European Society for Radiotherapy and Oncology (GYN GEC–ESTRO) formed a task group with the general aim to introduce a common target concept IGABT in vaginal cancer. In preparation of this task, the multicenter retrospective international image-based adaptive BRAchytherapy for Vaginal cancer (retroEMBRAVE) study was conducted to collect clinical data from patients with primary vaginal cancer that have been treated with 3D-image-guided adaptive brachytherapy.

The present study reports on the outcomes of the RetroEMBRAVE study.

## 2. Materials and Methods

RetroEMBRAVE is an international retrospective observational study involving five European centers that actively participate in the GYN GEC–ESTRO network. All participants have experience with state-of-the-art IGABT for gynecologic cancer and participate in the EMBRACE (an intErnational study on MRI-guided BRAchytherapy in locally advanced CErvical cancer) studies [10].

### 2.1. Patients

All patients treated in the five centers were consecutively included in the study if they fulfilled the following criteria: histologically confirmed primary vaginal cancer (squamous cell, adeno- or adenosquamous cell carcinoma); MRI at time of diagnosis; treatment with curative treatment intent with combined external beam radiotherapy EBRT (+/− concomitant cisplatin) and CT–MRI guided (Pulsed-dose rate (PDR) or High-dose rate (HDR)) IGABT, or in selected patients brachytherapy alone.

Patient, tumor, and treatment characteristics as well as outcome data related to disease status, survival and morbidity were collected. Data collection started April 2014 and closed in July 2017. Due to variation in commencement of image-guided BT in the participating centers, patients were treated between July 2001 and September 2016 with the majority of the patients (72%) treated after January 2009.

All patients were clinically staged according to the International Federation of Gynecology and Obstetrics (FIGO) criteria [11]. Clinical tumor size was based on gynecological examination and defined as the maximum diameter (in any direction) of the palpable or visible mass in the vagina. Pathological lymph nodes were defined as lymph nodes >1 cm in size on CT, MRI and/or FDG-positive lymph nodes on PET CT. Imaging for distant metastases followed institutional guidelines and included at least chest X-ray.

### 2.2. Treatment

The target volume for EBRT included the vagina, paravaginal space, cervix, parametria and pelvic lymph nodes, and, dependent on the location of the vaginal tumor and pathological lymph nodes, the para-aortic and inguinal lymph nodes. A total dose of 45.0–50.4 gray (Gy) was given in 1.7–2.0 Gy per fraction. In case of pathological lymph nodes, a nodal (sequential or simultaneous) boost was given to a total equivalent (EQD2_α/β10_) dose of 60–64 Gy.

**Figure 1:**
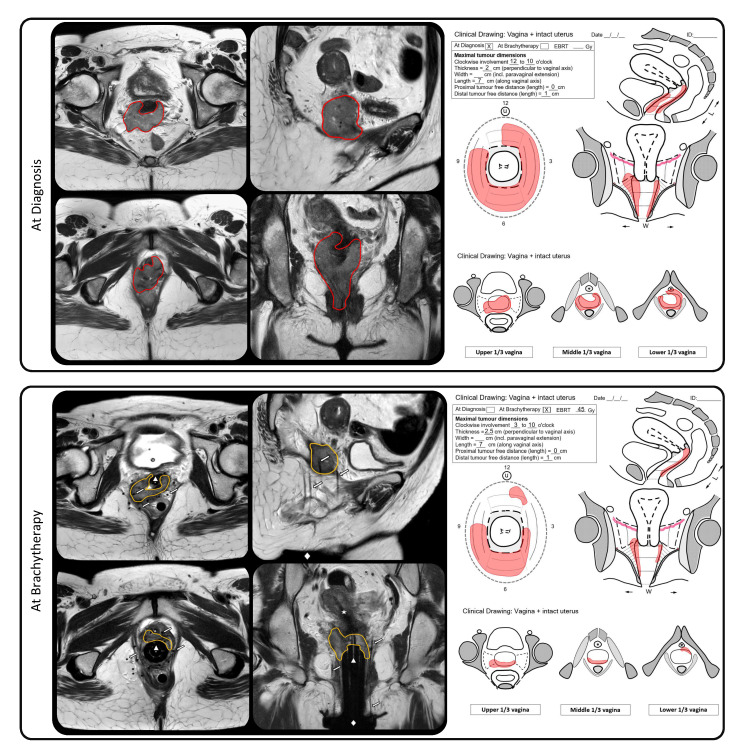
Example of a with MRI-guided brachytherapy treated patient with a bulky T2N0M0 primary vaginal cancer at diagnosis. The upper panel shows the MRI and clinical drawing at time of diagnosis. The lower panel shows the MRI, including the multichannel cylinder with an intrauterine tandem and paravaginal plastic needles inserted via a perineal template attached to the skin, and the clinical drawing at time of brachytherapy. In red, the tumor at time of diagnosis; in yellow, the residual tumor at time of brachytherapy representing the clinical target volume (CTV). The star represents the intra-uterine tandem, the triangle represents the multichannel cylinder, the diamond represents the perineal template and the arrows represent the interstitial paravaginal needles.

Image-guided brachytherapy was defined as the use of 3D imaging at the time of brachytherapy with MRI, MRI/CT or CT (with MRI at diagnosis). For the MRI/CT approach, aMRI with applicator (cylinder) in situ was conducted a few days before the application and matched with the CT on the applicator, at the time of brachytherapy. The CTV was delineated on the MRI and the organs at risk on the CT scan. During the period of patient inclusion, no vaginal-cancer-specific consensus guideline regarding target-volume definition and dose prescription was available. However, all centers prescribed—in accordance to their joint experience in cervical cancer [12,13] and initial experience in vaginal cancer [14,15]—the dose to a volume which was based on the residual tumor at the time of BT, as well as initial tumor extension at diagnosis. This volume included palpable/visible tumor by clinical examination, taking into account information from CT and/or MRI at BT and the MRI at the time of diagnosis. An example of a with MRI-guided-brachytherapy treated patient, including imaging and clinical drawings at the time of diagnosis and brachytherapy, is shown in Figure 1. Prescription dose to the target and dose constraints for the organs at risk (OAR) were according to individual practice in each center. All doses were converted to biologically equivalent dose in 2 Gy fractions (EQD2), using the linear quadratic model. An α/β ratio of 10 Gy was used for the target, and 3 Gy for organs at risk (OARs). For pulsed dose rate (PDR) a half-time of repair of 1.5 h was used for both the target and the OARs.

Patients were assessed for disease status and adverse effects, according to institutional guidelines, at regular intervals. In general, this was every 3 months in the first 2 years, every 6 months for the third year and annually thereafter.

### 2.3. Statistical Analyses

Local control (LC), pelvic control (PC), disease-free survival (DFS), overall survival (OS) and severe late morbidity were evaluated. Follow up for disease status was calculated as time from diagnosis until an event or last follow-up. Patients were censored at last follow-up or at the time of any disease recurrence during follow-up. Survival analysis for disease status was calculated by the Kaplan–Meier method, with 95% confidence intervals (95% CIs). Univariable analyses of risk factors for LC and DFS were conducted by Cox regression analysis. Significant risk factors (*p* ≤ 0.05) for the univariate analysis were entered in a multivariable Cox regression model. Late severe (grade 3 and higher) urogenital (GU), gastro-intestinal (GI) and vaginal morbidity were scored according to the common terminology criteria for adverse events version 3.0 (CTCAE, V3.0) and analyzed in crude numbers.

SPSS v.20 (IBM SPSS Statistics for windows, Version 20.0 Armonk, NY, USA: IBM Corp.) was used for statistical analysis.

## 3. Results

### 3.1. Patients Characteristics

A total of 148 patients were included. Patients characteristics are shown in Table 1. Median age at diagnosis was 63 years (interquartile range (IQR) 54–73). Twenty-eight (19%) patients had a tumor (T) classification 1, 79 (53%) had T2, 21 (14%) had T3 and 20 (14%) had T4. Forty-six (31%) patients had pelvic and/or inguinal lymph node metastases. The median maximum tumor diameter at diagnosis was 40 mm (IQR 30–60). The majority of patients (91%) had squamous cell carcinoma. Fifty (34%) patients previously underwent a hysterectomy because of a benign disease.

### 3.2. Treatment Characteristics

Treatment characteristics are shown in Table 2. Three patients with small T1 tumors were treated with brachytherapy alone. Concurrent weekly cisplatin was prescribed in 94 patients (64%). Most patients (65%) had one application (ranging from one to three) and were treated with PDR (78%). In the case of HDR, most patients received three fractions of 6–7 Gy. The median overall treatment time (OTT) was 48 (IQR 44–56) days.

### 3.3. Dose Volume Parameters

The median volume of the clinical target volume (CTV) was 17.6 cm^3^ (IQR 6.8–32.1), and the median D_90_ of the CTV was 80 Gy EQD2_α/β10_ (IQR 73.0–85.2). The median doses to the D_2cm3_ of the bladder, rectum, sigmoid and bowel were 64 Gy EQD2_α/β3_ (IQR 55–73), 64 Gy EQD2_α/β_ (IQR 57–68), 49 Gy EQD2_α/β_ (IQR 46–54) and 49 Gy EQD2_α/β_ (IQR 46–57), respectively. Doses for sigmoid and bowel were not reported in 40 (28%) and 87 (62%) of the patients. In the remaining patients, these OARs were located far from the target, resulting in no significant dose from the brachytherapy boost. In seven patients, information on dose-volume histogram (DVH) parameters could not be retrieved from older treatment planning systems.

### 3.4. Oncological Outcomes

**Figure 2:**
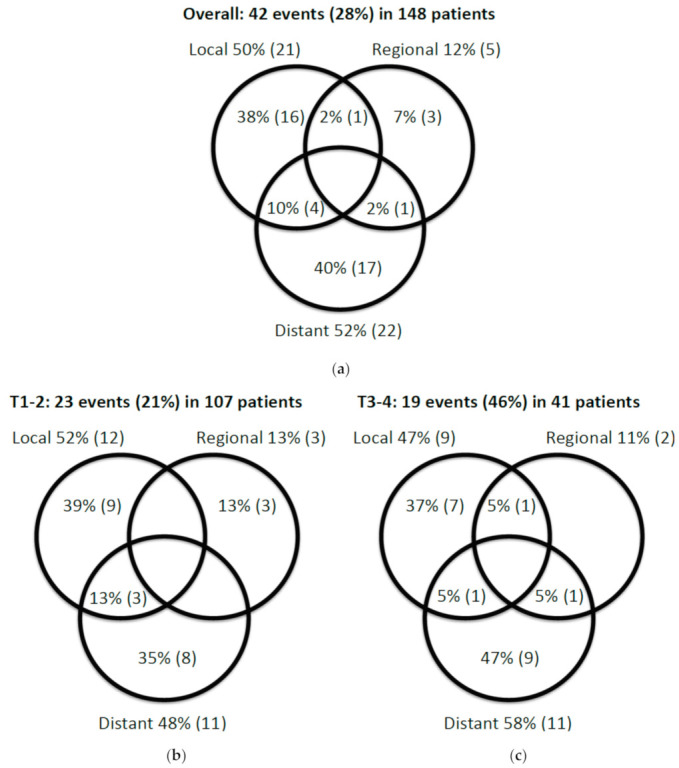
Venn diagrams for (**a**) local, regional and distant recurrences overall (*n* = 42); and (**b**,**c**) local, regional and distant recurrence according to T-classification (T1/T2 and T3/T4).

At a median follow-up of 29 months (IQR 25–57), 42 recurrences (local, regional and/or distant) had been reported, of which 16 were isolated vaginal recurrences (Figure 2a). Patients with T1/T2 tumor had an overall recurrence rate of 21% (23/107) and with T3/T4 tumors 46% (19/41). The pattern of recurrence was, however, similar in the two groups (Figure 2b,c).

**Figure 3:**
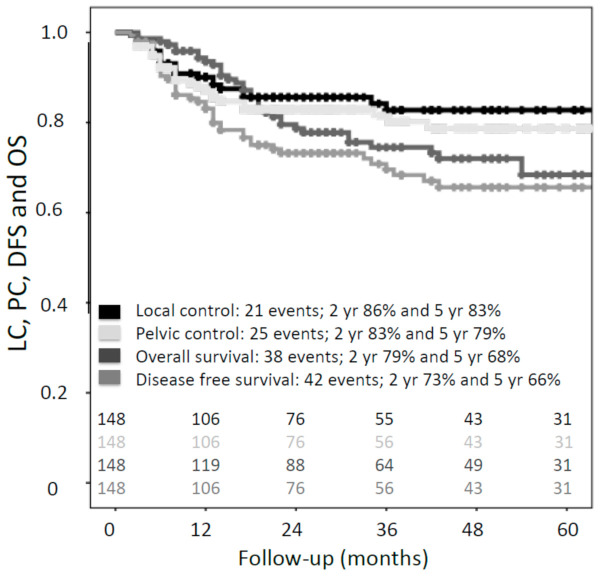
Kaplan–Meier estimates for local control (LC), pelvic control (PC), overall survival (OS) and disease-free survival (DFS) for the entire cohort (*n* = 148).

Actuarial two- and five-year local control (LC) were 86% (95% CI, 80–94) and 83% (95% CI, 76–90), respectively (Figure 3). Ninety percent (19/21) of all local recurrences occurred within the first two years of follow-up. Only two local recurrences occurred later, after 34 and 36 months, respectively. Actuarial two- and five-year pelvic control (PC) were 83% (95% CI, 77–90) and 79% (95% CI, 71–87), respectively (Figure 3). During follow-up, one patient treated with brachytherapy alone recurred regionally, and the remaining two patients did not have any recurrence. Two- and five-year disease-free survival (DFS) were 73% (95% CI, 65–81) and 66% (95% CI, 57–75), and overall survival (OS) was 79% (95% CI, 71–87) and 68% (95% CI, 59–77), respectively (Figure 3).

Twenty-five patients (17%) developed severe (grade ≥3) morbidity. In 23 patients, one grade-three/four event was diagnosed. In the remaining two patients, two severe events were diagnosed. The crude incidence of ≥ grade-three urogenital, gastro-intestinal and vaginal morbidity was 8.1%, 3.0% and 8.1%, respectively.

Only one grade-four event occurred, namely a life-threatening vesico-vaginal fistula. This patient had a bulky (6 cm) T2N0 tumor located in the lower third of the vagina. She was treated in 2008, with a combination of EBRT and concurrent cisplatin, followed by CT-guided brachytherapy. Due to the good response on EBRT, she was treated with an intracavitary only approach (mould) at time of brachytherapy. The fistula was diagnosed 2.5 years after treatment. She had no comorbidities and did not smoke. However, at the age of 37, she was treated for a vulvar Bowen disease with radiotherapy, and was thus partially re-irradiated.

No grade-five morbidity was seen. The most frequent severe morbidities were vesico-vaginal fistulas (*n* = 7) and complete vaginal obliteration (*n* = 10). Nine out of ten patients with complete vaginal obliteration had involvement of the lower (distal) third of the vagina at the time of diagnosis.

### 3.5. Prognostic Factors

**Figure 4:**
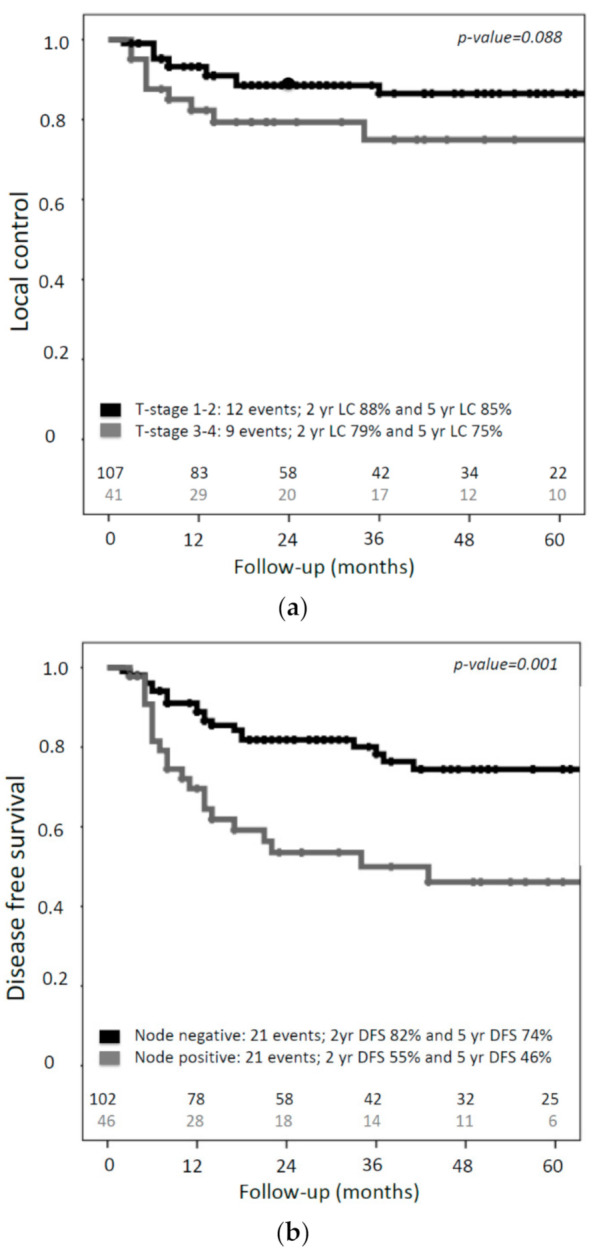
Kaplan–Meier estimates for (**a**) local control according to T-classification and (**b**) disease-free survival according to N-classification.

Univariable analysis of prognostic factors for LC showed a trend (*p*-value 0.088) towards better five-year local control in patients with a T1/T2 (85%; 95% CI, 78–94) versus T3/T4 tumor (75%; 95% CI, 59–90) (Figure 4a). Univariate analysis of prognostic factors for disease-free survival (DFS) showed that T-classification, maximum tumor diameter at diagnosis and lymph node status were associated with DFS (Table 3). In the multivariable analysis only lymph node status remained an independent prognostic factor for DFS (HR 2.24 (95% CI, 1.18–4.22); *p* = 0.013) (Table 3). Patients with lymph node metastases had a significantly lower two-year DFS, compared to lymph-node-negative patients (55% versus 82%) (Figure 4b).

**Figure 5:**
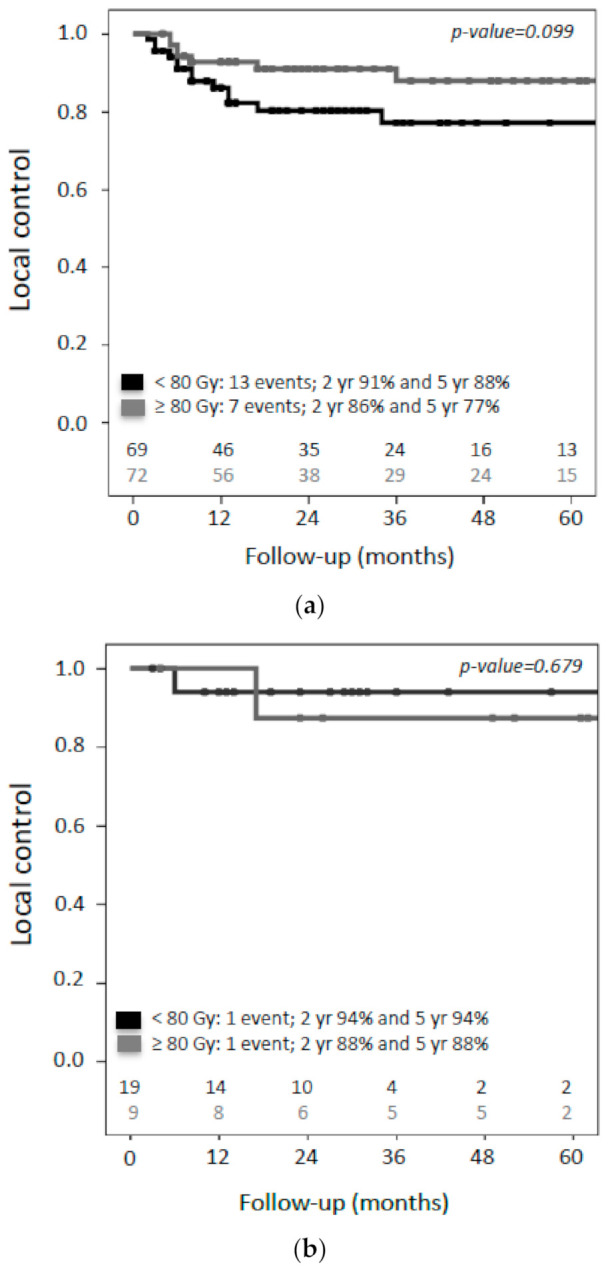

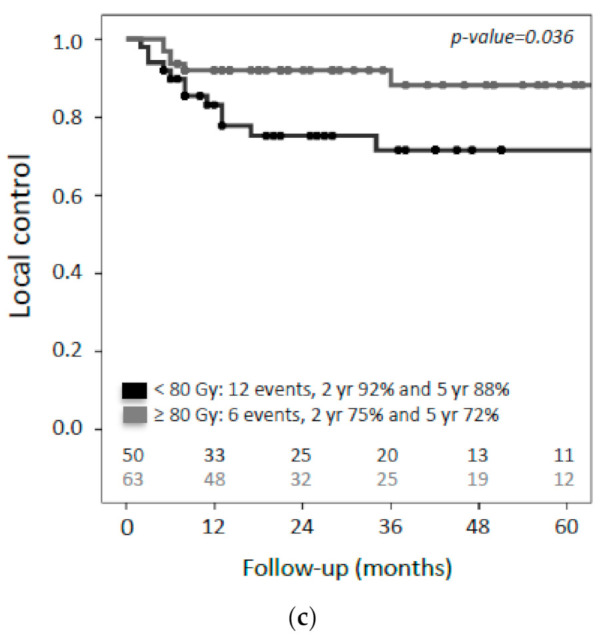
Kaplan–Meier estimates for local control according to dose to the D_90_ of the CTV (*n* = 141). Cutoff is the median dose (80Gy EQD2 _α/β10_). (**a**) Local control in the entire cohort, (**b**) local control in small (T1) tumors and (**c**) local control in advanced (T2–T4) tumors.

Analysis of the median delivered dose to the CTV D_90_ (minimal dose to 90% of the target volume) of the CTV showed a trend in better local control in favor of patients who received more than 80 Gy EQD2_α/β10_ This difference was, however, not statistically significant (*p*-value = 0.099) (Figure 5a). When analyses were conducted according to T-classification, no dose-effect was found in the group of patients with small (T1) tumors (Figure 5b). In patients with advanced (T2–T4) tumors, improved local control was seen if the CTV received more than 80 Gy EQD2_α/β10_ (92% versus 75% at two years, *p*-value = 0.036) (Figure 5c). Due to the low number of events, no meaningful multivariate analysis could be performed for local control.

## 4. Discussion

This study investigated the outcomes of definitive radiotherapy with CT/MRI-based radiotherapy in a large retrospective, observational cohort of consecutively included patients with primary vaginal cancer. Two-year local and pelvic control were 86% and 83%, respectively. In patients with advanced (T2–T4) tumors, a significantly better local control was found if patients received more than 80 Gy EQD2_α/β10_ in the clinical target volume at time of brachytherapy (92% versus 75% at two years, *p* = 0.036). T-stage, maximum tumor diameter at diagnosis and lymph node status were associated with DFS in univariable analysis. However, only lymph node status remained an independent prognostic factor for DFS (HR, 2.24; 95% CI, 1.18–4.22; *p* = 0.013), with two-year DFS for lymph-node-positive patients 55% versus 82%, respectively. Crude incidence of overall (GI, GU and vaginal) severe (≥ grade 3) morbidity was 17%, of which vaginal and urogenital morbidities were most frequently observed.

Introduction of the GEC–ESTRO recommendations for MRI-guided IGABT in locally advanced cervical cancer [12,13], resulted in an improved therapeutic ratio with increased local control and simultaneous reduced late morbidity [9]. More recently this has found its way into the new ICRU-89 report, in collaboration with GEC–ESTRO, for prescribing, recording, and reporting brachytherapy for cervical cancer [8]. Given these encouraging clinical results, individual centers have also implemented IGABT as standard treatment for vaginal cancer [14,15,16,17,18,19]. In agreement with our results, previous monocentric experiences showed high two-year local control rate of 92% (range 82–93) [14,15,16,17,18,19]. Although comparison between studies from the radiograph-based era and modern studies employing IGABT is challenging due to the retrospective nature, case mix differences and number of included patients, results of the IGABT era seem to compare favorable, especially in the group of patients with large tumors [3]. In advanced (T3/T4) tumors, the difference in pelvic control between radiograph and CT/MRI-based brachytherapy is 63% versus 79% at five years, respectively [20,21,22,23,24,25]. Only one small study analyzed the differences in morbidity between radiograph- and CT/MRI-based brachytherapy planning for primary vaginal cancer [19]. This study included 72 patients with primary vaginal cancer; the patients who received CT/MRI-guided brachytherapy had less grade ≥2 GI/GU morbidity compared to patients that were treated with radiograph-based brachytherapy [19]. Noteworthy, morbidity in the historical radiograph-based studies was reported with varying level of detail, and especially for vaginal morbidity, it probably was underestimated.

There are several explanations for the improved outcomes presented in our study, compared to historical series. First, in the majority of patients, MRI was used for delineation and planning at time of brachytherapy. Since MRI is the preferred imaging modality for visualization of pelvic tumors due to its superior soft tissue contrast, the chance of a geographical miss is reduced, as compared to radiograph-based brachytherapy [3]. Second, a combined intracavitary/interstitial (IC/IS) approach was used more frequently (55%) in this study, as compared to the radiograph-based studies [20,21,22,23,24,25]. The use of interstitial needles generally leads to improved coverage of the target [3]. Although in all earlier radiograph-based studies interstitial needles were applied, the percentage of patients treated with IC/IS approach was less than in our study [20,21,22,23,24,25]. Third, the dose prescribed to the CTV at the time of brachytherapy was higher. Safe dose-escalation in the tumor and dose-de-escalation in the OARs by highly conformal radiotherapy is only possible with the use of volumetric imaging modalities, especially MRI, and advanced brachytherapy techniques with the use of needles in large tumors. Finally, concurrent cisplatin as a radiosensitizer has become standard in patients with advanced tumors. Apart from evidence from randomized trials in cervical cancer, a large National Cancer Database (NCDB) analysis in primary vaginal cancer patients showed that cisplatin was an independent prognostic factor for improved overall survival [5]. In our study, 64% of the patients received chemotherapy, while, in the older studies, only a small proportion of the patients received chemotherapy.

Despite the large number of included patients for this rare tumor, this retrospective study has several limitations. First, there was no published target concept during the period of patient inclusion. However, the participating centers were active in IGABT for cervical cancer and all adopting these basic concepts into their treatment, with a presumed high agreement in the clinical target volume for dose prescription. Second, different volumetric imaging modalities at brachytherapy were allowed, as long as a diagnostic MRI was available. This very much reflects the clinical practice where a transition is made also with regard to availability of MRI compatible IC/IS applicators. Third, the median follow-up (FU) of 29 months may seem short. However, the majority of local recurrences take place in the first 24 months [24,26]. An explanation for the shorter FU is that part of these patients are specifically referred from further away to our centers for these more complex BT procedures, and these patients are more prone to be lost to FU. Finally, the retrospective design has inherent limitations, especially regarding the reporting of morbidity.

The presence of lymph node metastasis was an independent prognostic factor for DFS. A finding that is in line with experience in cervical cancer, where this led to incorporation of the nodal status into the new FIGO 2018 classification. This underscores the importance of adequate imaging at the time of diagnosis with MRI and (PET–)CT for staging purposes [27,28]. In addition, use of MRI for target contouring and treatment planning at time of BT is essential, to adopt the IGABT strategy as proposed in the recently published (GYN) GEC–ESTRO/ACROP recommendations for IGABT in primary vaginal cancer [29]. In the present study, a minority (28%) of the patients were treated with CT-only guided brachytherapy. Use of CT at brachytherapy can be applied in specific conditions, provided that a MRI at diagnosis and accurate documentation of the clinical findings at the time of diagnosis and brachytherapy is available. For this purpose, standardized cartoons that illustrate the anatomy of the vagina and surrounding organs at the time of diagnosis and brachytherapy are very useful and should be routinely implemented in clinical practice (Figure 1) [29].

At present, clear evidence regarding the optimal dose that should be prescribed in patients with primary vaginal cancer is lacking. Most evidence has come from the results of IGABT in locally advanced cervical cancer within the EMBRACE studies [13]. In only one study from the radiograph-based era with 91 patients, a trend (*p* = 0.055) towards improved pelvic control was found in patients that were treated with a total dose of at least 70 Gy [22]. The authors concluded that the optimal dose for primary vaginal cancer probably ranges between 70 and 80 Gy EQD2_α/β10_, which is also in agreement with the recommendations from the American Brachytherapy Society (ABS) for interstitial brachytherapy for vaginal cancer [30]. Notably, the dose was prescribed to a point (5 mm depth or at the vaginal surface) and not to a volume [22]. In our study, the dose was prescribed to a clinical target volume (CTV), and patients with T2–T4 tumors had a higher local control with more than 80 Gy EQD2_α/β10_ in the CTV D90, at the time of brachytherapy (92% versus 75% at two years). Despite the limitations of this study, this result is in line with the impact of IGABT demonstrated in cervical cancer. For the small group of patients (*n* = 28) with T1 tumors, a very high local control rate was found and, thus, no dose effect relationship could be established (Figure 5a). To draw more firm conclusions regarding the optimal dose for primary vaginal cancer, the present results have to be confirmed in a larger prospective study. Delineation of the different target volumes at the time of brachytherapy (GTV-T_res_, CTV-T_HR_ and CTV-T_IR_), according to the recently published GYN GEC–ESTRO/ACROP recommendations for a common target concept in primary vaginal cancer, is essential to be able to evaluate the results in a multicenter setting [29]. A prospective multicenter study (EMBRAVE) is being initiated, to evaluate this new target concept in primary vaginal cancer and will provide clinical reference data for the evaluation of dose–effect relationships. Within this study, doctor- and patient-reported morbidity will also be evaluated, since systematically and prospectively evaluated data are lacking thus far.

## 5. Conclusions

Our large contemporary multicenter study confirms the findings of previous small, monocentric studies regarding the role of image-guided brachytherapy in primary vaginal cancer, demonstrating good local and pelvic control of 86% and 83% at two years, respectively. Especially in T2–4 tumors an improved local control was found compared to historic radiograph-based studies. In these larger T2–4 tumors a dose of ≥80 Gy (cumulative dose in EQD2_α/β10_) to the clinical target volume was associated with a higher local control in univariate analysis. Fortunately, these high local control rates did not appear to lead to higher severe late toxicity, presumably due to the possibility of maximal sparing of the organs at risk with 3D planning. These findings warrant, however, prospective validation using the recently published common target concept and methodology for dose reporting in primary vaginal cancer.

## Figures and Tables

**Table 1:** Patient and tumor characteristics (*n* = 148).

T-classification	
1	28 (19%)
2	79 (53%)
3	21 (14%)
4	20 (14%)
N-classification	
0	102 (69%)
1	46 (31%)
Histology	
Squamous cell carcinoma	134 (91%)
Adenocarcinoma	8 (5%)
Adenosquamous carcinoma	6 (4%)
Maximum tumor diameter at diagnosis	
≤4 cm	77 (52%)
>4 cm	71 (48%)
Vaginal involvement at diagnosis	
Upper third	26 (18%)
Middle third	16 (11%)
Lower third	38 (26%)
Upper two-thirds	14 (9%)
Lower two-thirds	14 (9%)
Entire vagina	23 (16%)
Unknown	17 (11%)

T = tumor; N = node; M = metastasis.

**Table 2:** Treatment characteristics (*n* = 148).

Concurrent cisplatin	
No	54 (36%)
Yes	94 (64%)
EBRT technique	
APPA/box	8 (5%)
3DCRT IMRT/VMAT	82 (57%) 55 (38%)
Dose rate	
PDR	115 (78%)
HDR	33 (22%)
Number of applications	
1	100 (68%)
2	36 (24%)
3	12 (8%)
Imaging at time of BT	
CT	42 (28%)
MRI	77 (52%)
MRI/CT ^#^	29 (20%)
BT technique	
Intracavitary	67 (45%)
Interstitial +/− intracavitary	81 (55%)

^#^ MRI with applicator in situ was used for preplanning and contouring of the target in combination with a computer tomography (CT) for the definitive application and planning. APPA = anterior–posterior/posterior–anterior; EBRT = external beam radiotherapy; 3DCRT = 3D conformal radiotherapy; IMRT = intensity modulated radiotherapy; VMAT = volumetric-modulated arc therapy; PDR = pulsed dose rate; HDR = high dose rate; BT = brachytherapy; MRI = magnetic resonance imaging.

**Table 3:** Multivariable analysis of prognostic factors for DFS.

Variable	Univariate Analysis			Multivariate Analysis		
	HR	95% CI	*p*-Value	HR	95% CI	*p*-Value
Age						
<63 vs. ≥63	0.72	0.38–1.36	0.288			
Chronic disease						
Yes vs. No	0.63	0.30–1.31	0.215			
Hysterectomy						
Yes vs. No	1.02	0.55–1.89	0.947			
Tumor-stage						
T1/T2 vs. T3/T4	2.27	1.23–4.16	0.008 *	1.65	0.86–3.15	0.134
Tumor diameter at diagnosis						
≤4 cm vs. >4 cm	1.92	1.03–3.57	0.041 *	1.42	0.73–2.73	0.301
Lymph node metastases						
Yes vs. No	2.75	1.50–5.03	0.001 *	2.24	1.18–4.22	0.013 *
Concomitant chemotherapy						
Yes vs. No	1.72	0.38–1.36	0.311			
CTV volume at brachytherapy						
<17.6 cm^3^ vs. ≥17.6 cm^3^	1.10	0.55–2.18	0.794			

* Statistically significant; HR = hazard ratio; CI = confidence interval; vs. = versus; CTV = clinical target volume.

## Data Availability

All data associated with this study are present in the paper.
